# Nek5: a new regulator of centrosome integrity

**DOI:** 10.18632/oncotarget.5139

**Published:** 2015-08-10

**Authors:** Suzanna L. Prosser, Andrew M. Fry

**Affiliations:** Lunenfeld-Tanenbaum Research Institute, Mount Sinai Hospital, Toronto, Ontario, Canada

**Keywords:** Nek5, centrosome integrity, ciliopathy, microcephaly, primordial dwarfism

The centrosome is the major microtubule-organising centre of an animal cell. It dictates the arrangement of a radial microtubule array in interphase and bipolar microtubule spindle in mitosis. In differentiated cells, the centrosome also promotes growth of the primary cilium, a sensory antenna-like structure that transduces external stimuli into intracellular signals. The physiological importance of the centrosome is highlighted by the detection of mutations in many genes encoding centrosome components in human developmental disorders, including microcephaly (MCPH), primordial dwarfism (PD), and a range of syndromic ciliopathies [1].

MCPH, a disease characterized by small brain size, and PD, which manifests as whole body growth retardation, are due to reduced cell numbers that result from loss of the relevant stem cell populations. So how do centrosomes contribute to maintenance of stem cell numbers? Centrosomes are structurally complex organelles consisting of two substructures: a centriole pair that is surrounded by pericentriolar material (PCM). The PCM contains proteins responsible for microtubule nucleation and anchoring, while the centrioles provide the scaffold onto which the PCM is assembled. During mitosis centrosomes exhibit increased microtubule nucleating capacity due to PCM expansion. This recruitment of additional PCM components is crucial for spindle assembly and, if it does not occur, disorganised spindles arise that trigger mitotic arrest, cell death and/or aneuploidy. Hence, defective spindle organization in the stem cell niche can trigger cell loss through senescence or apoptosis. However, stem cell number is also determined by spindle orientation with symmetric divisions driving self-renewal and maintenance of stem cell number. Spindle defects that favour asymmetric divisions lead to premature differentiation and reduction of the progenitor pool. Spindle orientation depends upon attachment of astral microtubules from centrosomes to the cell cortex, centrosome defects can disrupt these attachments thereby promoting spindle asymmetry and reduction of the stem cell pool.

Mutations in two key PCM scaffold components, CDK5RAP2 (MCPH3) and pericentrin, cause MCPH and PD, respectively [2, 3]. CDK5RAP2 is required for recruitment of γ-tubulin complexes - the sites of microtubule nucleation - to the PCM, attachment of centrosomes to spindle poles, and formation of astral microtubules. Similarly, pericentrin functions to organize the PCM and recruits proteins involved in microtubule nucleation and anchoring, including γ-tubulin and CDK5RAP2. Depletion of pericentrin disrupts nucleation in mitosis leading to disorganised spindles, chromosome misalignment, premature sister chromatid separation and aneuploidy. Astral microtubule formation is also perturbed leading to spindle misorientation. Mutations in a further MCPH locus (MCPH2, encompassing the *WDR62* gene), prevents centrosomal recruitment of CDK5RAP2 and γ-tubulin, underlining the importance of centrosome integrity in preventing this disorder [4]. In addition to profound mitotic consequences, loss of centrosome integrity induces a p38-p53-p21-dependent G1-arrest or senescence [5]. Therefore, loss of centrosome integrity through mutation of PCM components, such as CDK5RAP2 and pericentrin, contributes to MCPH and PD via cell cycle arrest, cell death and loss of spindle symmetry, all contributing to depletion of progenitor stem cells.

We have recently described a new regulator of centrosome integrity, the NEK5 protein kinase [6]. NEK5 localises to centrosomes and its depletion led to reduced centrosomal γ-tubulin, pericentrin, and CDK5RAP2 in both interphase and mitosis. This caused reduced microtubule nucleation, mitotic delay, altered spindle geometry and chromosome segregation errors. It remains to be determined how NEK5 contributes to centrosome integrity. It could phosphorylate key PCM components, such as CDK5RAP2 or pericentrin. Alternatively, it could regulate other proteins required for their recruitment. Given the severity of human disorders that result from loss of centrosome integrity, dissecting the mechanism of action of NEK5 in cell and animal models is likely to yield important insights into these diseases.

Loss of centrosome integrity also contributes to kidney and liver cyst development, a pathological hallmark of many ciliopathies that arise from primary cilium defects. PCM proteins have not been directly linked to ciliopathies, however depletion of pericentrin reduced targeting of intraflagellar transport proteins and polycystin-2 to the centrosome, and inhibited ciliogenesis [7]. Cells carrying mutant pericentrin also have abnormal cilium-dependent signalling pathways. Hence, centrosome integrity appears vital for the centrosome to act as a structural support and coordinator of protein trafficking for cilium assembly and function. In mouse models, misorientation of cell division also contributes to ciliopathy phenotypes. Given the role of pericentrin in spindle positioning, its mutation may contribute to these phenotypes through spindle misorientation. The role of NEK5 at the primary cilium remains to be studied. However, its localization to the base of the cilium and upregulated expression in response to transcriptional programmes that drive ciliogenesis hint at a potential role. Indeed, it is provocative that other kinases related to NEK5, including NEK1 and NEK8, are mutated in ciliopathies. Direct sequencing of Nek5, and its substrates once identified, could therefore reveal novel mutations not only in ciliopathy patients but also other developmental disorders, including MCPH and PD, for which the genetic basis of disease remains to be found.
